# Integrated High-Throughput Centrifugal Microfluidic Chip Device for Pathogen Detection On-Site

**DOI:** 10.3390/bios14060313

**Published:** 2024-06-19

**Authors:** Shuyu Lu, Yuanzhan Yang, Siqi Cui, Anyi Li, Cheng Qian, Xiaoqiong Li

**Affiliations:** 1 School of Medical Technology, Beijing Institute of Technology, 5 South Zhongguancun Street, Haidian District, Beijing 100081, China; lushuyu@bit.edu.cn (S.L.); 3120215950@bit.edu.cn (Y.Y.); 3220225270@bit.edu.cn (S.C.); 3120236079@bit.edu.cn (A.L.); cheng95@bit.edu.cn (C.Q.); 2 Beijing Key Laboratory for Separation and Analysis in Biomedicine and Pharmaceuticals, Beijing Institute of Technology, 5 South Zhongguancun Street, Haidian District, Beijing 100081, China; 3 Key Laboratory of Convergence Medical Engineering System and Healthcare Technology, the Ministry of Industry and Information Technology, 5 South Zhongguancun Street, Haidian District, Beijing 100081, China

**Keywords:** centrifugal microfluidic chip, nucleic acid extraction, lamp, paraffin valve, hydrophobic valve, siphon valve, Coriolis force, device

## Abstract

An integrated and high-throughput device for pathogen detection is crucial in point-of-care testing (POCT), especially for early diagnosis of infectious diseases and preventing the spread of infection. We developed an on-site testing platform that utilizes a centrifugal microfluidic chip and automated device to achieve high-throughput detection. The low-power (<32 W), portable (220 mm × 220 mm × 170 mm, 4 kg) device can complete bacterial lysis, nucleic acid extraction and purification, loop-mediated isothermal amplification (LAMP) reaction, and real-time fluorescence detection. Magnetic beads for nucleic acid adsorption can be mixed by applying electromagnetic fields and centrifugal forces, and the efficiency of nucleic acid extraction is improved by 60% compared to the no-mixing group. The automated nucleic acid extraction process achieves equivalent nucleic acid extraction efficiency in only 40% of the time consumed using the kit protocol. By designing the valve system and disc layout, the maximum speed required for the centrifugal microfluidic chip is reduced to 1500 rpm, greatly reducing the equipment power consumption and size. In detecting *E. coli*, our platform achieves a limit of detection (LOD) of 10^2^ CFU/mL in 60 min. In summary, our active centrifugal microfluidic platform provides a solution for the integration of complex biological assays on turntables, with great potential in the application of point-of-care diagnosis.

## 1. Introduction

The outbreaks of infectious diseases caused by pathogens have increased the demand for POCT for public health monitoring. Recently, miniaturized molecular diagnostic platforms have been designed for on-site testing and various microfluidic technologies, with the inherent benefits of compact nature, small reagent consumption, and precise and automatic fluid control. They can be assembled in various functional designs, and have been developed for pathogen detection [1,2,3]. Among them, the centrifugal microfluidic platform is an ideal technology that has been developed in recent years for POCT DNA detection [4,5]. The centrifugal microfluidic chip focuses more on the ability to integrate nucleic acid extraction and analysis [6,7]. Integrated chips have the advantage of composite functionality and can also reduce the amount of chips used and the time and operations required for detection. The integrated chip has good sealing, which is conducive to avoiding contamination that may occur when the reagents are transferred and improving the anti-interference ability of on-site pathogenic microbial detection. Although quantitative real-time PCR (qPCR) is the gold standard for nucleic acid testing, it requires extremely accurate and rapid temperature control, which places extremely high demands on the equipment. To eliminate the necessity of the thermal cycler and simplify operation, the isothermal amplification method is proposed, in which amplification can be accomplished at a constant temperature within a short time [8,9]. Integrating centrifugal microfluidic chips with LAMP reactions enables parallel detection and has the advantages of small sample volume requirements, simple reagent loading process, fast speed, high throughput, and high efficiency [10,11,12].

The integration of bacterial lysis, nucleic acid extraction and purification, and nucleic acid detection to establish a centrifugal-driven microfluidic chip system is currently the focus of research on integrated detection equipment. Traditional microfluidic chips rely on bulky syringe pumps, valve actuators, and complex tubing systems for transferring and placing liquids, which makes their processing technology complex, resulting in insufficient efficiency during testing and prolonged testing time [13]. Compared with traditional pressure-driven methods, centrifugal microfluidic chips have the advantage of higher potential for automation [14]. However, most work to achieve the graded opening of the fluid control circuitry, step up the speed during centrifugation, or maintain centrifugal motion for long periods [15]. This ultimately leads to excessively high maximum centrifugal speeds and high motor power consumption, taking away from the original intent of instant detection. Therefore, to achieve an integrated automatic detection process, it is necessary to consider the design of chip valves. Centrifugal microfluidics were clarified into two categories: passive centrifugal microfluidics and active centrifugal microfluidics [16]. However, passive valves still have some limitations in terms of performing complex logical operations or time sequencing with more steps, which limits their application on centrifugal microfluidic chips. Centrifugal microfluidic chips in recent years have tended to have a flexible combination of passive/passive valves and active/ciliated valves, finding a balance between stability and simplicity [17]. These fluid control circuits have achieved good results.

Furthermore, nucleic acid extraction is the initial and necessary step of nucleic acid testing, and the quality of nucleic acid determines the result. However, due to the complexity and time-consuming nature of sample preprocessing and nucleic acid extraction, the DNA is usually extracted and purified directly outside the chip and added by the micro pipettor. Lander developed an automatable rotationally driven microfluidic device for the isolation of SARS-CoV-2 RNA. However, sample enrichment using Nanotrap magnetic virus particles was conducted off-disc, reducing the automation of the detection process [18]. In addition, some studies have integrated traditional nucleic acid extraction methods into detection systems but inevitably complicated the equipment, increased sample processing time, and introduced amplification inhibitors. A study developed a portable genetic analyzer with an integrated centrifugal disc, but the extraction efficiency of magnetic beads for nucleic acid was relatively low, and the sample pre-treatment was time-consuming [19]. The nucleic acid adsorption process based on centrifugal microfluidic chips often employs a membrane [20]/silica column [21]/magnetic bead [22,23] approach. With more efficient solid–liquid mixing efficiency, magnetic beads have been widely used in the on-chip nucleic acid adsorption process. In the project, centrifugal force was skillfully utilized to control the opening of the siphon valve circuit. However, the magnetic beads were bound by the centrifugal force during the adsorption process and could not be actively mixed, which reduced the extraction efficiency and made the process time-consuming.

This study proposes an integrated and fully automated centrifugal microfluidic chip for the rapid detection of pathogenic microbials. The proposed strategy of active mixing or active collecting of magnetic beads allows nucleic acid extraction on the centrifugal microfluidic chip with the same efficiency as that of the kit while reducing the time consumed by 60%. The automated nucleic acid extraction process of the centrifugal microfluidic chip achieves the same nucleic acid extraction efficiency in 40% of the time required by the kit protocol. By introducing paraffin valves, hydrophobic valves, siphon valves and Coriolis force structure, the on-chip fluid control is accurately accomplished, while the maximum centrifugal rotational speed is controlled at only 1500 rpm. The low-power (<32 W) and portable (220 mm × 220 mm × 170 mm, 4 kg) automation unit contains five modules: centrifugation, heating, electromagnetic field, fluorescence detection, and control system. Within the automated device, the variety flows in the centrifugal microfluidic chip for nucleic acid extraction and the LAMP reaction is fully automated. Our centrifugal microfluidic chip requires only a simple reaction reagent and sample filling, and the remaining steps of the assay are fully automated. We tested this protocol with *E. coli* and achieved detection within 60 min. Detection of *E. coli* in a centrifugal microfluidic chip showed that our system achieved the LOD of 10^2^ CFU/mL for genomic DNA.

## 2. Materials and Methods

### 2.1. Fabrication of Centrifugal Microfluidic Chips

**Figure 1:**
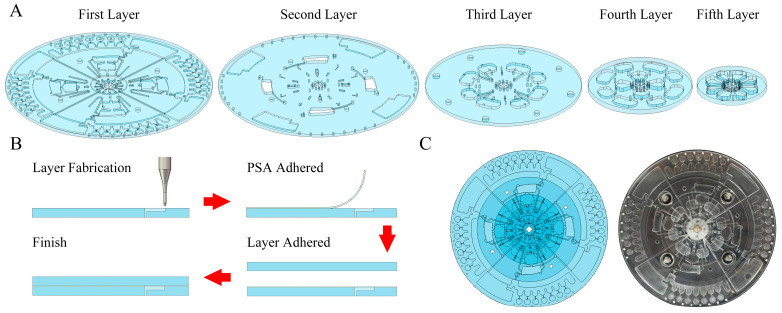
Micropattern and fabrication of centrifugal microfluidic chip. (**A**) Multiple layers of centrifugal microfluidic chip. (**B**) Fabrication of PMMA layer and assembly of centrifugal microfluidic chip. (**C**) Scheme and photo of centrifugal microfluidic chip.

The 3-dimensional (3D) model of the centrifugal microfluidic chip was designed using Solidworks 2020. Polymethyl methacrylate (PMMA) has been widely used in the fabrication of centrifugal chips due to PMMA’s excellent light transmission and biocompatibility. Five PMMA layers (Figure 1A) with diameters of 136 mm, 136 mm, 90 mm, 60 mm, and 40 mm were processed using a high-speed precision engraving and milling machine to form functional structures on the front and back of the PMMA layers. These PMMAs were rinsed twice using ddH_2_O and dried in an oven at 60 °C for 3 h. Low-temperature paraffin wax with a melting point of 40 °C was selected and melted at 120 °C; then, 1 μL was taken and added to the paraffin valve and subsequently cooled and solidified at room temperature. The hydrophobic valves and siphon valves were treated using MesoPhobic-2000 (Wuhan Biotechnology Co., Ltd. Wuhan, China) and 0.2% (*V*/*V*) Tween-20 (dissolved in anhydrous ethanol), respectively, and dried naturally at room temperature. Finally, five layers of PMMA chips and four layers of pressure-sensitive adhesive (PSA, 3M 9969Solventum, St Louis, USA) were sequentially pressed together to form the overall centrifugal microfluidic chip. Screws were fixed onto the edge of the chip to avoid air bubbles that may be generated by the PSA under different temperature conditions. The schematic and photo of the whole machining process are shown in Figure 1B,C.

### 2.2. Development and Assessment of Automated Devices

**Figure 2:**
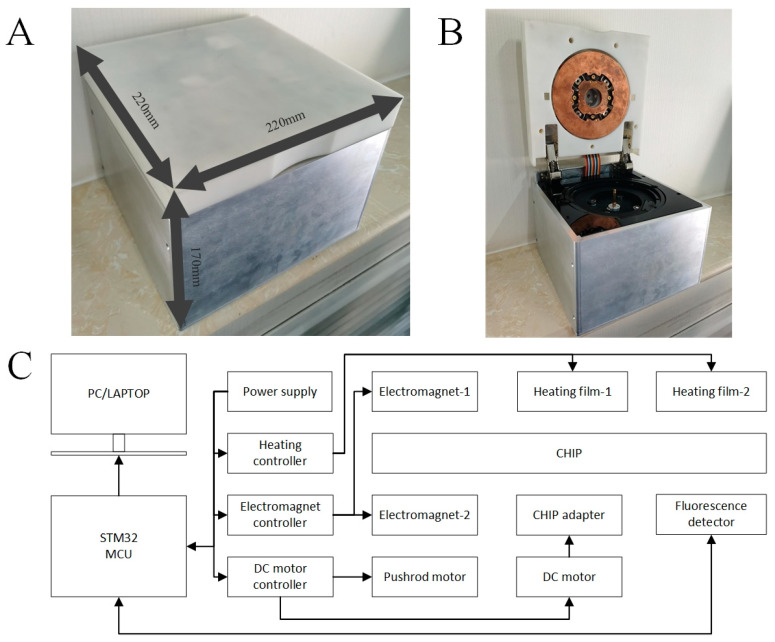
Automatic device. (**A**) External dimensions of the automation device. (**B**) The automation device from the user’s operational viewpoint. (**C**) The schematic diagram of the automation device.

We designed an integrated device for automated work on centrifugal microfluidic chips (Figure 2A,B). For rapid deployment and detection on-site, automation devices should have the advantage of being portable and low-power. The dimensions of the automation device are 220 mm × 220 mm × 170 mm and the weight is 4 kg. The automated device contains a centrifugal module, a heating module, an electromagnetic field module, a fluorescence detection module, and a control system. The centrifugal module consists of a direct current (DC) motor (GA36Y-3530-12100, Xinyongtai, Shenzhen, China) with a photoelectric switch. The heating module consists of two pairs of PI (Polyimide) heating membranes and purple copper disks. The electromagnet module consists of eight miniature low-power electromagnets. The fluorescence detection module utilizes a miniature fluorescence detector (MNS-MFD-470, MONAISI, Suzhou, China) with excitation and emission wavelengths of 470 mm and 525 mm. The control module consists of data analysis software, an STM32 microcontroller, power supply, a heating controller (TEC207M, SenseFuture, Shenzhen, China), an electromagnet controller, a DC motor controller, and a pushrod motor. The centrifugation program, the heating program, the magnetic bead mixing/collection program, the fluorescence detection program, and the result-sending program are loaded into the microcontroller unit (MCU).

Fluid control relies on precise speed control. We set multiple rotational speeds within the maximum speed of the centrifugal chip to examine the linear relationship between the actual rotational speed and the duty ratio of pulse width modulation (PMW). The PWM is generated through the MCU. The actual speed of the centrifugal chips was measured with a hand-held tachometer (DLY-2301; DELIXI, Hangzhou, China). We fitted the data linearly using rotational speed as the horizontal coordinate and the duty ratio of PWM as the vertical coordinate.

A low-noise fluorescence detector with an accurate response is essential for plotting amplification curves. We set up a concentration gradient of sodium fluorescein solution (0 μmol/L, 0.2 μmol/L, 0.4 μmol/L, 0.6 μmol/L, 0.8 μmol/L, and 1 μmol/L) for the purpose of checking the working performance of the fluorescence detector. During fluorescence response signal acquisition, we rotated the centrifugal chip to spin at 60 rpm. After adding ddH_2_O to all amplification chambers, the fluorescence response signals were measured for one week of centrifugal chip spinning using the ADS1256 module on the MCU. The background noise of the detector was calculated from these fluorescence response signals. Subsequently, we fitted the data linearly with the fluorescein sodium concentration as the horizontal coordinate and the fluorescence response signal as the vertical coordinate.

After initiating automated pathogenic microbial detection, the lysis and amplification reactions have stringent requirements for the accuracy and stability of temperature control. We measured the effect of temperature control for the lysis reaction (56 °C) and the LAMP reaction (65 °C), respectively. Temperature sensors were installed in four lysis chambers and four amplification chambers. We used the MCU to record the temperature sensor for 10 min at 2 Hz when the predetermined temperature is reached. The temperature records were then evaluated, and time–temperature curves were plotted.

Finally, we evaluated the power consumption of the automation device using a power meter, and the results were used to confirm that the automation device had the advantage of low power consumption.

### 2.3. Nucleic Acid Extraction and LAMP Reaction Program

We used the product of centrifugation of *E. coli* in place of samples of pathogenic microbials obtained from the environment. First, 90 μL ATL buffer (QIAGEN, Hilden, Germany) and 10 μL proteinase K (QIAGEN, Hilden, Germany) were used as lysis reagents. The nucleic acid adsorption reagent consisted of 10 μL of magnetic beads and 140 μL of MB buffer (QIAGEN, Hilden, Germany). A 160 μL sample of ddH_2_O was used as the wash buffer, so disturbance or mixing of the magnetic bead clusters needed to be strictly avoided during the washing process. We used 147 μL of LAMP cocktail (Table S1) as nucleic acid elution buffer. Each amplification chamber contained 3.2 μL of LAMP primer mixture (Table S2).

Here, we describe the reaction process of detecting actual samples in a centrifugal microfluidic chip. (i) We resuspended the *E. coli* precipitate using the lysis reagent. (ii) The lysis process involved heating the sample for 20 min at 56 °C. (iii) The lysis process product, MB buffer, and magnetic beads were thoroughly mixed and incubated for 1 min to adsorb nucleic acids. (iv) We collected the magnetic beads to remove the nucleic acid adsorption waste solution. (v) We kept the collected magnetic beads, added washing buffer, and held for 10 s. (vi) We kept the collected magnetic beads and removed the bead wash waste solution. (vii) The LAMP cocktail was mixed with the magnetic beads and incubated for 1 min to elute the nucleic acids. (viii) We collected the magnetic beads, transferred the LAMP cocktail with nucleic acids, and pre-distributed the LAMP cocktail. (ix) The LAMP primer mixture was added to the pre-distributed LAMP cocktail with nucleic acids. (x) A 35 min LAMP reaction was conducted at 65 °C.

The bead mixing or collection required in the above steps was achieved by providing centrifugal force and an electromagnetic field to the centrifugal microfluidic chip using our automated device. To achieve bead mixing, we applied alternating rotation (±100 rpm, 2 Hz) and an electromagnetic field to the centrifugal microfluidic chip. Alternating rotation generated a liquid flow to drive the magnetic beads for mixing. Electromagnetic fields helped the beads to overcome gravity and friction for smoother bead movement. To achieve magnetic bead clustering, our magnetic bead clustering procedure consisted of accelerating the centrifugal microfluidic chip from 0 to 300 rpm and holding it for 10 s.

### 2.4. Reagent Preloading

Before the chip began its automated work, 140 μL of MB buffer, 10 μL of magnetic beads, 160 μL of ddH_2_O, and 147 μL of LAMP cocktail needed to be added to the corresponding storage chambers within the chip, and 3.2 μL of LAMP primers mixture was added to each amplification chamber. The above addition inlets were subsequently sealed using a transparent plate-sealing film (YA0245, Solarbio, Beijing, China).

### 2.5. Assessment Procedures of Detection

An experiment was conducted to assess the enhancement of nucleic acid extraction efficiency by magnetic bead mixing. The same volume of lysis product, MB buffer, and magnetic beads were added to the chambers of the experimental and control groups. During nucleic acid adsorption and elution, the magnetic beads of the experimental group were mixed, while the magnetic beads of the control group remained stationary. Finally, we measured the concentrations of both groups of nucleic acids in MicroplateReader (Cytation3, Biotek, WA, USA).

The efficiency of nucleic acid extraction was compared between the automated protocol and the kit protocol using 100 μL of *E. coli* (10^8^ CFU/mL). In the automated protocol, nucleic acid extraction reagents were preloaded. The lysis system was added to a centrifugal microfluidic chip, followed by a 25 min automated nucleic acid extraction process. The kit protocol was then based on a standardized nucleic acid extraction process. Nucleic acid extraction products from both protocols were evaluated using a qPCR instrument (Gentier 96, TIANLONG, Xi’an, China). The reagent composition and primers for the 20 μL qPCR system are shown in Table S3 and Table S4, respectively.

To validate the LAMP primers, we set up LAMP experiments in centrifuge tubes with a reaction system of 25 μL of LAMP system (the reagent composition is shown in Table S5). The target of the experimental group was used as the product of the automated nucleic acid extraction protocol, and the target of the control group was used as ddH_2_O. We performed the LAMP reaction using a qPCR instrument for 35 min at 65 °C. The feasibility of the LAMP primers was assessed by observing whether the experimental group could amplify normally versus whether the control group had no obvious fluorescence signal.

After validating the LAMP primers, we assessed the effectiveness of the LAMP cocktail as a nucleic acid elution buffer. Positive controls were made using 100 μL of 108 CFU/mL of *E. coli* as the sample, and negative controls were made without adding any *E. coli*. We tested all of the reactions on the chip by simulating the process in a centrifuge tube and used 147 μL of LAMP cocktail for nucleic acid elution. Finally, 16.8 μL of LAMP cocktail was mixed with 3.2 μL of LAMP primer mixture for a 35 min, 65 °C LAMP reaction in the qPCR instrument. The effectiveness of the LAMP cocktail as a nucleic acid eluent was evaluated by observing whether the experimental group could amplify normally versus whether the control group had no obvious fluorescent signal.

Based on previous experiments, we clarified the automated process and the composition of the reagents required for the reaction. Subsequently, we validated the ability to perform nucleic acid extraction and LAMP reactions coherently on the chip. We added 3.2 μL of LAMP primer mixture to each of the seven amplification chambers in a channel. Automated nucleic acid extraction and LAMP reactions were performed on the chip using 100 μL of *E. coli* (10^8^ CFU/mL). We recorded the fluorescence signals of the seven amplification chambers and plotted the LAMP reaction curves in the result analysis software.

The ability of the chip to perform the complete process of *E. coli* detection was confirmed by the analysis of amplification curves. Finally, we designed four concentrations of *E. coli* samples to evaluate the LOD. In this case, four concentrations of samples were obtained with 0, 10, 10,000, and 100,000-fold dilutions using *E. coli* with 10^8^ CFU/mL. Samples of 100 μL of four concentrations of *E. coli* were injected into each of the four sample channels for testing. We performed a linear fit to obtain a quantitative calibration curve of concentration–fluorescence signal values using the fluorescence signal value at 24 min as the vertical coordinate and the concentration of the bacterial solution as the horizontal coordinate. The LOD of *E. coli* was calculated according to the 3-σ principle.

## 3. Results

### 3.1. Design and Process of Centrifugal Microfluidic Chips

**Figure 3:**
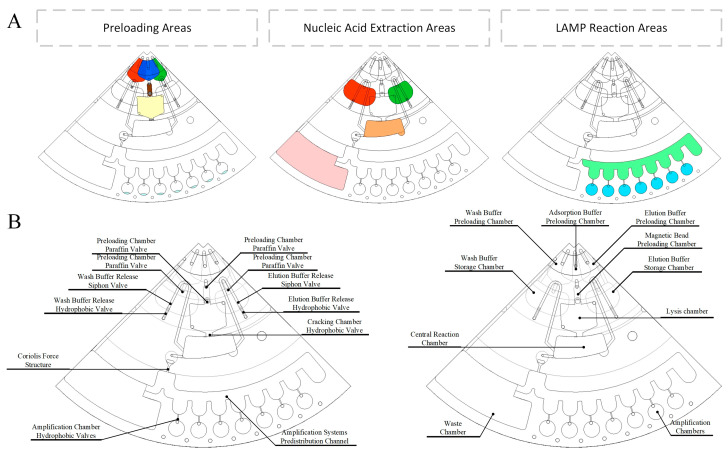
Centrifugal microfluidic chip. (**A**) Area division in centrifugal microfluidic chip. (**B**) Schematic diagram of valves and chambers in centrifugal microfluidic chip.

The centrifugal microfluidic chip for on-site multiple detection of pathogenic microbial has three functional areas (Figure 3A): preloading areas, nucleic acid extraction areas, and LAMP reaction areas. To fully utilize the centrifugal potential energy, the three functional zones and the chambers within the functional areas were essentially arranged outward along the radius. The paraffin valves, hydrophobic valves, siphon valves, Coriolis force structure, and pre-distribution channel were designed to be used for accurate liquid movement and quantitative distribution (Figure 3B).

### 3.2. Flow Control of the Centrifugal Microfluidic Chip

**Figure 4:**
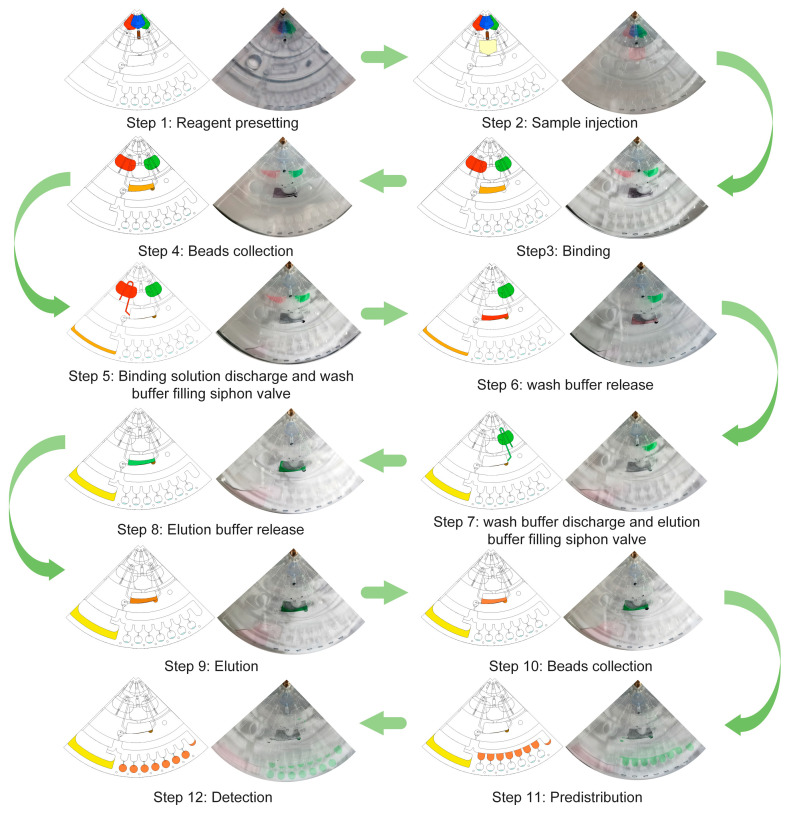
Flow control of the centrifugal microfluidic chip. Step 1: Indicator reagents for adsorption buffer(blue area in the schematic figure), magnetic beads(brown area in the schematic figure), wash buffer(red area in the schematic figure), and elution buffer(green area in the schematic figure) are preloaded into the preloading chamber. Indicator reagents for primers are preloaded in the amplification chamber. Step 2: Indicator reagents for lysis buffer with samples(yellow area in the schematic figure) are injected into the lysis chamber. Step 3: Indicator reagents for wash buffer and elution buffer are transferred to the storage chamber. The indicator reagents for the binding solution (lysis product, adsorption buffer, magnetic beads) are released into the central reaction chamber and the magnetic beads are mixed using centrifugal force and electromagnetic field. Nucleic acids are adsorbed to the magnetic beads. Step 4: The magnetic beads form agglomerates. Step 5: The adsorbate is transferred to the waste chamber and the wash buffer is allowed to fill the siphon valve. Step 6: The wash buffer is released and the magnetic beads are kept in state. Step 7: The binding solution is transferred to the waste chamber and the elution buffer is allowed to fill the siphon valve. Step 8: The elution buffer is released. Step 9: The centrifugal force and electromagnetic field are used to mix the magnetic beads with the elution buffer. Nucleic acids are released from the magnetic beads into the elution buffer. Step 10: The magnetic beads form agglomerates. Step 11: The elution buffer enters the pre-dispensing channel. Step 12: The elution buffer is mixed with the indicator for the primers.

The complete process of nucleic acid extraction and LAMP reaction assay consisted of eight solution transfer steps (1#~8#) and twelve states (step 1~step 12). 1# Preloading and lysis: The preloading chambers and the amplification chambers were injected with the reaction reagents (Figure 4, step 1); the lysis chambers were injected with the samples (Figure 4, step 2); and subsequently, the lysis chamber was heated at 56 °C for 20 min to lyse the sample and melt the paraffin valve. 2# Nucleic acid adsorption: The MB buffer, magnetic beads and lysis products were transferred to the central reaction chamber at 300 rpm; ddH_2_O and LAMP cocktails were transferred from the preloading chamber to the respective storage chambers; and the magnetic beads were mixed in the central reaction chamber using alternating rotation (± 100 rpm, 2 Hz) and an electromagnetic field (Figure 4, step 3). 3# Nucleic acid adsorption discharge: Using 300 rpm and electromagnetic force, the magnetic beads in the central reaction chamber were gathered to the side away from the outlet (Figure 4, step 4); using −600 rpm, the nucleic acid adsorption was discharged to the waste chamber, while allowing the ddH_2_O to breach the hydrophobic valve downstream of the staging chamber. 4# Bead Wash: The chip was allowed to rest and we filled the siphon valve downstream of the hydrophobic valve with ddH_2_O (Figure 4, step 5); we then used −300 rpm to release the ddH_2_O into the center reaction chamber (Figure 4, step 6). 5# Bead Wash Solution Discharge: We drained the bead wash waste solution into the waste chamber using −800 rpm while allowing the LAMP cocktail to break through to the waste chamber where it resided. It was located in the hydrophobic valve downstream of the staging chamber. 6# Nucleic acid elution: The chip was allowed to rest and ddH_2_O filled the siphon valve downstream of the hydrophobic valve (Figure 4, step 7). The ddH_2_O was released into the center reaction chamber using −300 rpm (Figure 4, step 8); the magnetic beads were mixed in the center reaction chamber using alternating rotation (±100 rpm) and an electromagnetic field (Figure 4, step 9). 7# Pre-dispensing: Using 300 rpm and electromagnetic force, the magnetic beads inside the center chamber were collected to the side away from the downstream outlet (Figure 4, step 10); LAMP pre-mixed with nucleic acids was quantified in the pre-dispensing channel using 1000 rpm (Figure 4, step 11). 8# Amplification system configuration and amplification reaction: using 1500 rpm, the LAMP pre-mixed with nucleic acids, i.e., the LAMP cocktail, was centrifuged into the amplification chamber to form the amplification system and start the LAMP reaction (Figure 4, step 12).

### 3.3. Performance of Automation Integration Devices

To realize fully automated nucleic acid extraction and LAMP amplification reactions in a centrifugal microfluidic chip, we built an automated device. The automated device has functions including centrifugation, heating, electromagnetic field, and fluorescence detection. We designed each functional module compactly and tested its working performance.

**Figure 5:**
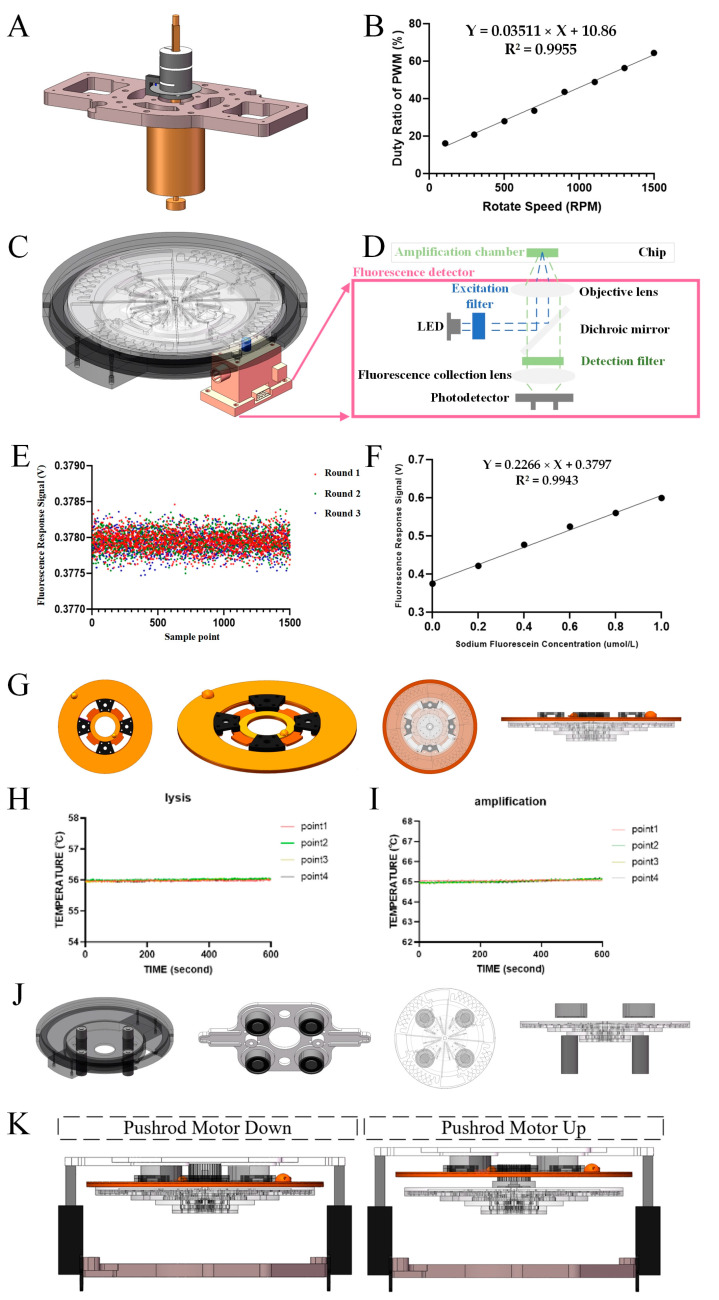
Automatic device design. (**A**) Schematic diagram of centrifugation module. (**B**) Precise rotational speed control based on PWM. (**C**) Fluorescence detector is installed on the chip slot for fluorescence scanning detection of the rotating chip. (**D**) Schematic diagram of the fluorescence detector showing the folded optical path and optics. (**E**) The background noise of the fluorescence detector was obtained by repeating the fluorescence test three times. (**F**) Corresponding curves of fluorescence response signals and fluorescein sodium concentration. (**G**) Schematic diagram of the heating module, and schematic diagram of chip-heating module in the heating step. (**H**) Real-time temperature curves inside the four lysis chambers. (**I**) Real-time temperature curves inside the four amplification chambers. (**J**) Electromagnet module’s schematic diagram, and the schematic diagram of the chip–electromagnet module in the magnetic bead control step. (**K**) The working status of the actuator motor: the pushrod motors rise during the heating processes and detection processes, and the pushrod motors lower during other processes.

Although we ultimately determined that eight specific speeds would be sufficient to drive the fluid for the complete process, the centrifugal module was needed to achieve precise speed control during valve design to determine the actual operating speed of the valve. The centrifugal module for spinning centrifugal microfluidic chips is shown in Figure 5A. The integrated aluminum alloy holder held the DC motor, photoelectric switch, and chip adapter. Precise rotational speed control was achieved by applying PWM (Figure 5B). The PWM duty ratio had a good linear relationship with rotational speeds between 100 and 1500 rpm. The instantaneous error for each target speed was within ±3 rpm. We used photoelectric switches and DC motors to achieve the alternating rotation of the centrifugal chip, which was designed to oscillate the central chamber with the magnetic beads in an area centered on an electromagnet.

The working position and schematic diagram of the fluorescence detection and the chip are shown in Figure 5C and Figure 5D, respectively. The folded optical path makes the detector miniaturized. The excitation light (470 nm) emitted by the LED is converged in the amplification chamber of the chip through the excitation filter, dichroic mirror, and objective lens. The emitted light (525 nm) from the amplification chamber is converged on the surface of the photodetector by means of an objective lens, a dichroic mirror, a detection filter, and a fluorescence collection lens. As shown in Figure 5E, a fluorescence scanning assay was repeated three times after adding ddH_2_O inside the amplification chamber, and the standard deviation of the background signal was calculated to be 0.16 mV, indicating that the fluorescence detection module has a low background noise. Concentration–fluorescence response signal curves (Figure 5F) were plotted for the test results of sodium fluorescein solutions with gradient concentrations (0 μmol/L, 0.2 μmol/L, 0.4 μmol/L, 0.6 μmol/L, 0.8 μmol/L, and 1 μmol/L) in the chip. The results showed that the fluorescence detection module had a good linear response to changes in fluorescence intensity.

The heating module is shown in Figure 5G, where two copper disks with PI heating films are simultaneously fixed to the polyimide structure. These two copper violet disks faced the lysis chamber and amplification chamber inside the centrifugal microfluidic chip, respectively, and the copper disks were in contact with the chip during heating. Temperature monitoring was performed for 10 min (2 Hz) in four lysis chambers and four symmetrically positioned amplification chambers. The temperature inside all four chambers was maintained at 56 ± 0.1 °C during testing (Figure 5H). The temperature inside all four chambers was maintained at 65 ± 0.1 °C during the assay (Figure 5I). This demonstrates the stable and consistent temperature control of the automated device for both lysis and amplification reactions.

As shown in Figure 5J, the electromagnet module consisted of eight electromagnets. Four of the electromagnets were fixed on the chip slot where they directly faced the central reaction chamber. The other four electromagnets were fixed on an integrated flat plate together with the heating module, and these four electromagnets also directly faced the central reaction chamber.

The heating module was in contact with the centrifugal microfluidic chip. To ensure that the chip rotated properly, we implemented distance control between the heating–electromagnet assembly and the chip (Figure 5K). Thus, the automated device pulled the heating–electromagnet assembly in contact with the chip using pushrod motors during the heating processes of the lysis and amplification reaction. During the fluorescence detection processes, which included the liquid drive and amplification reactions, the pusher motor lifted the heater–electromagnet assembly and moved it away from the chip. Finally, we used a power meter to measure the power of the automated device when performing the full test, which showed a power consumption of 32 W.

### 3.4. Evaluation of Nucleic Acid Extraction and LAMP Reaction Systems

We performed absorbance tests (600 nm) on cultured *E. coli* using a MicroplateReader. We used *E. coli* (10^8^ CFU/mL) as the sample in the experiments to evaluate the effects of nucleic acid extraction, LAMP reaction, and full detection. We used *E. coli* (4 × 10^8^ CFU/mL) for the evaluation of the effect of magnetic bead mixing on nucleic acid extraction.

**Figure 6:**
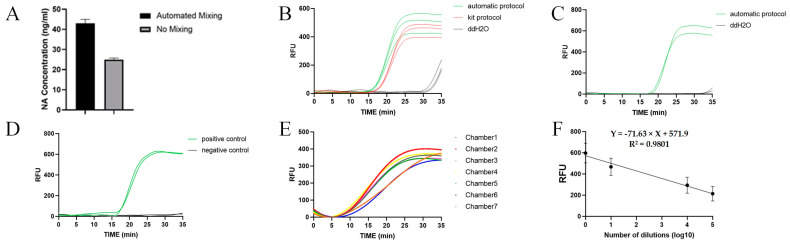
Evaluation of nucleic acid extraction and LAMP reaction systems. (**A**) Automated mixing increased nucleic acid extraction efficiency by 60% relative to the unmixed control. (**B**) The automated nucleic acid extraction efficiency was comparable to that of the kit’s standard process. (**C**) The primer set designed for the LAMP reaction correctly amplified fragments of the *E. coli* genome. (**D**) The LAMP cocktail allowed for nucleic acid elution. (**E**) Tests of the automated complete process on *E. coli* results. (**F**) Correspondence curve between nucleic acid concentration and fluorescence response signal.

To achieve higher nucleic acid extraction efficiency, we designed an automated magnetic bead mixing or collection procedure. The lysis products of 100 μL of *E. coli* (4 × 10^8^ CFU/mL) were added to two chambers, respectively. As shown in Figure 6A, the concentration of the nucleic acid extraction products was 40 ng/mL when the magnetic bead mixing or collection procedure was used and 25 ng/mL when the magnetic bead mixing or collection procedure was not used. The ratio of the concentrations of the two was 1.6.

The nucleic acid extraction efficiency of the automation on the chip is also one of the properties we are interested in. By using the same *E. coli* samples, nucleic acid extraction was performed on the chip and in the centrifuge tube, respectively. The automated nucleic acid extraction products obtained from the automatic protocol were subjected to qPCR together with the nucleic acid extraction products obtained from the kit protocol. The results of qPCR are shown in Figure 6B. The average CT values of the automated and kit protocols were 17 and 18, respectively, which proved that the nucleic acid extraction efficiency of the automated protocol was slightly better than that of the kit protocol.

We experimented with primers for the LAMP reaction using the *E. coli* genome from the automated protocol (Figure 6C). Positive controls had a mean CT value of 18.3, and negative controls had no significant increase in fluorescence intensity. The experimental results proved that LAMP primers can be used to amplify the *E. coli* genome. We experimented with LAMP cocktails as nucleic acid elution buffers and used these LAMP cocktails in LAMP reactions (Figure 6D). The positive group with the *E. coli* genome exhibited a mean CT value of 17.2, while the negative control group showed no significant increase in fluorescence intensity.

We performed detection experiments on *E. coli* samples on the chip to evaluate the feasibility and sensitivity of the chip for *E. coli* detection. The results for 100 μl of *E. coli* (10^8^ CFU/mL) are shown in Figure 6E, with a mean CT value of 14.3 (CV = 7%) obtained from seven chambers. In sensitivity experiments, four concentrations of *E. coli* were detected in 35 min, and we plotted a correspondence curve with nucleic acid concentration using the fluorescence response voltage at 24 min (Figure 6F). Based on the 3-σ principle, we calculated that the LOD of this centrifugal microfluidic chip for *E. coli* was 10^2^ CFU/mL.

## 4. Discussion

The transfer of reagents is fully automated; otherwise, an operational burden is created [24]. Automated reagent transfer relies on integrated valve design. The valves shown in Table 1 demonstrate that the multiple valves integrated into the centrifugal chip enable fluid control while avoiding the use of bulky valves. The paraffin valve is an active valve with a good seal before melting. We provided paraffin valves upstream and downstream of the reagent preloading chamber and the lysis reaction chamber. The additional heat generated by lysis was utilized to open the paraffin valve, avoiding the use of another heater and reducing the amount of passive valve. By connecting a hydrophobic valve and a siphon valve in series downstream of the reagent storage chamber, we combined the valve conditions for reagent release processes with those for waste solution discharge. Specifically, while the waste solution of the previous step was discharged, the reagent for the next step was selected, but not immediately released. When the waste solution was completely discharged, the reagents for the next step were released by centrifugation at a low speed or by keeping stationery. We controlled the flow of solution into the waste chamber or pre-distribution channel using the Coriolis force structure. This is a functional structure that selects the direction of flow by changing the direction of centrifugation. The pre-distribution channel allowed the LAMP cocktail, which contained nucleic acids, to be dispensed. Users can perform multiple LAMP reactions from the same sample. The above design directly helps to fully differentiate between the eight solution transfer steps by only requiring a maximum speed of 1500 rpm. According to Table 1, these examples of centrifugal microfluidic chips with nucleic acid extraction and amplification for the detection of pathogenic microbials generally require a maximum rotational speed greater than 2500 rpm. However, motors with high rotational speed are large and consume large amounts of power, which is not conducive to the portable design of the automation device and its on-site use. While extraction-free LAMP reactions and active valves have been shown to reduce the maximum operating speed of centrifugal microfluidic chips, they may reduce the reaction efficiency and increase the detection time [25].

In our on-site centrifugal microfluidic chip for the multiplexed pathogenic microbial detection system, *E. coli* was used as the sample for performance testing. We used the lysis reagent from the QIAGEN Magattract HMW DNA Kit, as this allowed for more efficient lysis of *E. coli* and minimized the inhibition of amplification. The results in Figure 6B,C demonstrate that the designed on-chip nucleic acid extraction process did not produce amplification inhibition for either the qPCR or the LAMP reaction.

Insufficient mixing of magnetic beads was responsible for the inefficient and time-consuming nucleic acid adsorption and elution [17]. In a centrifugal microfluidic chip, the magnetic beads tend to be deposited on the wall furthest from the axis in the chamber due to centrifugal force. At the same time, due to the small size of the microfluidic chip chamber, the deposited magnetic beads cannot be adequately mixed due to the restricted liquid flow. To overcome these problems, this study provides a magnetic bead control method based on alternating rotation and electromagnetic field control. By increasing the mixing or collection of the magnetic beads, efficient nucleic acid adsorption and elution were thus achieved. None of the cases with magnetic bead mixing within Table 1 compared the effect of magnetic bead mixing or lack thereof on the nucleic acid extraction efficiency, and we introduced experiments to assess the role of magnetic bead mixing in centrifugal microfluidic chips. According to the results of Figure 6A, we found that the magnetic bead mixing step enhanced the efficiency of nucleic acid extraction by 60% compared to the unmixed control. This suggests that adequate mixing of the magnetic beads in the lysis product is important to verify the efficiency of nucleic acid extraction. We confirmed that our automated protocol requires only 40% of the time of the kit protocol to achieve the same nucleic acid extraction efficiency (Figure 6B).

We completed the whole process of nucleic acid extraction and LAMP reaction on a centrifugal microfluidic chip. The results were consistent with the previous nucleic acid extraction experiments and LAMP reaction detection experiments. As shown in Figure 6E, the automated system realized the detection of the *E. coli* genome. We performed the whole process of nucleic acid extraction and LAMP reaction in a single channel, and the average CT value of the amplification curves in seven chambers was 14.3 (CV = 7%). These data support the evaluation of the consistency and stability of our centrifugal microfluidic chip and automated device against *E. coli*. In further sensitivity experiments, we derived a linear curve of dilution versus fluorescence intensity, as shown in Figure 6F. We calculated the LOD of the system to be 10^2^ CFU/mL. According to Table 1, our automatic detection system can achieve close detection sensitivity at lower rotational speeds. Our centrifugal microfluidic chip has the potential for multi-target detection because we designed the chip with four channels, each corresponding to seven assay chambers, and we verified the consistency and stability of each chamber by using *E. coli*.

## 5. Conclusions

Herein, we report a centrifugal microfluidic chip detection system for on-site multiplexed pathogenic microbial detection. We integrated nucleic acid extraction with LAMP reaction in a centrifugal microfluidic chip. An isolated pathogenic microbial detection space was created on-site. The automated device and the centrifugal microfluidic chip enable fully automated *E. coli* detection. The system has the advantages of portability (220 mm × 220 mm × 170 mm, 4 kg) and low power consumption (<32 W). Automated detection eliminates the requirement for operational expertise and reduces the cost of labor and time. We use the additional heat generated by the lysis to open the paraffin valve. We also designed the flow control system using a variety of passive valves and Coriolis force structures. These efforts reduced the burden of valve design and ultimately resulted in a reduction in the maximum speed of the entire process to 1500 rpm. We attempted to control the magnetic beads through electromagnetic fields and centrifugal force to achieve efficient bead mixing and collection within the reaction chamber. Compared with the control group without mixing magnetic beads, the experimental group that mixed magnetic beads with lysed samples achieved a 60% increase in nucleic acid extraction efficiency. The complete on-chip nucleic acid extraction process achieved the same extraction efficiency in 40% of the time of the kit protocol. The overall duration of our centrifugal microarray was 60 min, and we achieved a detection limit of 10^2^ CFU/mL for *E. coli*. In the future, we will integrate multiple LAMP detection technologies to achieve higher throughput and more sensitive on-site detection of pathogenic microbials.

## Figures and Tables

**Table 1:** Overview of centrifugal microfluidics for pathogenic microbial detection.

Sample to Answer	Nucleic Acid Extraction Material	Bead Mixing (if Bead Was Used)	Valve	Maximum Rotational Speed	Time	Number of Sample Channel/Detection Chamber of a Channel	LOD	Ref.
Yes	Magnetic beads	Yes	Paraffin valves, hydrophobic valves, siphon valves, Coriolis force structure	1500 rpm	1 h	4/7	*E. coli:* 10^2^ CFU/mL	This study
Yes	No nucleic acid extraction	-	Hydrophobic valves, capillary valves, siphon valves	3000 rpm	40 min	2/10	*E. coli:* 10^2^ CFU/mL *S. aureus*, *P. mirabilis*, *P. aeruginosa:* 10^2^ CFU/mL *S. typhimurium*: 10^3^ CFU/mL	[26]
Yes	FTA	-	Silicone oil layer, check valves	3000 rpm	1 h	8/1	*S. typhimurium*: 10 CFU/mL	[20]
Yes	No nucleic acid extraction	-	Pneumatic valves, wax valves	1500 rpm	70 min	4/4	*Salmonella:* 5 × 10^−3^ ng/μL	[25]
Yes	Silicone membrane	-	Pneumatic siphon valves, capillary valves, Coriolis force structure	2500 rpm	1 h	2/4	25 *Escherichia* or 40 *Salmonella*	[27]
No	Magnetic beads	Yes	Laser valves	3000 rpm	30 min	8	Only SARS-CoV-2 RNA extraction	[28]
Yes	Magnetic beads	No	Siphon valves, ball valve (magnet attracted), capillary valves, Coriolis force structure	3600 rpm	1 h	2/8	-	[17]
Yes	GF/F	-	Siphon valves, capillary valves, Coriolis force structure	5000 rpm	1.5 h	2/9	LAMP MPF, BDB, CDF: 10^3^ copies/μL FHV: 10^4^ copies/μL PCR FHV, MPF, CDF: 10^3^ copies/μL BDB: 10^3^ copies/μL	[19]
Yes	Magnetic beads	Yes	Capillary valves	3000 rpm	~50 min	5/8	-	[24]

## Data Availability

The data presented in this study are available upon request from the corresponding author.

## Supplementary Materials

The following supporting information can be downloaded at: https://www.mdpi.com/article/10.3390/bios14060313/s1, Table S1. Reagent composition of 147 μL LAMP cocktail; Table S2. Primer sequence for the LAMP reaction; Table S3. Reagent composition of 20 μL qPCR system; Table S4. Primer sequence for the qPCR reaction; Table S5. Reagent composition of 25 μL LAMP reaction.

## Abbreviations

LOD: limit of detection; LAMP: loop-mediated isothermal amplification; POCT: point-of-care testing; qPCR: quantitative PCR; 3D: 3-dimensional; PMMA: polymethyl methacrylate; DC: direct current; MCU: microcontroller unit; PWM: pulse width modulation; PSA: pressure-sensitive adhesive.
