# Supplementary material for: Integrated High-Throughput Centrifugal Microfluidic Chip Device for Pathogen Detection On-Site
Source: Biosensors (Basel). 2024 Jun 19;14(6):313. doi: 10.3390/bios14060313 (PMC11202104; doi:10.3390/bios14060313)
Supplement: Supplementary file 1 [file biosensors-14-00313-s001.zip › biosensors-3052914-supplementary.pdf]

Theoretical and simulation analysis for the microfluidic chip

First, we analyze the pressure ΔP caused by surface tension in channels such as hydrophobic valves, and obtain its expression according to the Young-Laplace formula:

$$\Delta P = -\gamma \times \left(\frac{COS\ \theta_L + \text{COS}\ \theta_R}{w} + \frac{COS\ \theta_T + \text{COS}\ \theta_B}{h} \right)$$

γ is used for the surface tension coefficient of the liquid in the channel, θ_L, θ_R, θ_T, θ_B are the contact angles of the left, right, top and bottom of the channel, w and h represent the width and height of the valve channel.

Secondly, the liquid in the channel receives the action of centrifugal force, and the pressure P_{cen} generated in the valve is:

$$P_{cen} = \frac{1}{2} \times \rho \times \omega^2 \times ({r_o}^2 - {r_i}^2)$$

The ρ is the density of the liquid, ω is the angular velocity of the chip centrifugation, r_o is the maximum liquid radius, r_i is the minimum liquid radius.

When the liquid in the valve is balanced under the action of centrifugal force and surface tension, $\Delta P = P_{cen}$, the calculation formula of the burst speed ω is as follows:

$$\omega = \sqrt{\frac{2 \times \gamma}{\rho \times ({r_o}^2 - {r_i}^2)} \times \left(\frac{|COS\ \theta_L| + |\text{COS}\ \theta_R|}{w} + \frac{|COS\ \theta_T| + |\text{COS}\ \theta_B|}{h} \right)}$$

The parameters are predetermined, γ=72.75×10^{-3} N/m, r_o = 26mm, r_i = 22mm, the contact angle of hydrophobic valves to 110°. We use this theoretical formula to guide the design of two different hydrophobic valves to achieve the step release of wash buffer and elution buffer. The width and height of the trap for washing reagents and elution buffer release are designed to be 0.4mm*0.2mm and 0.3mm*0.1mm, the burst speed ω are 592 and 790RPM respectively.

We performed fluid simulations using COMSOL software to guide valve design. While two-dimensional simulations may be missing the effect of valve height on valve opening, they can still help us quickly obtain information on the ordering of different valve burst speeds. As shown in the figure, we used fluid simulation to verify the order of the burst speed.

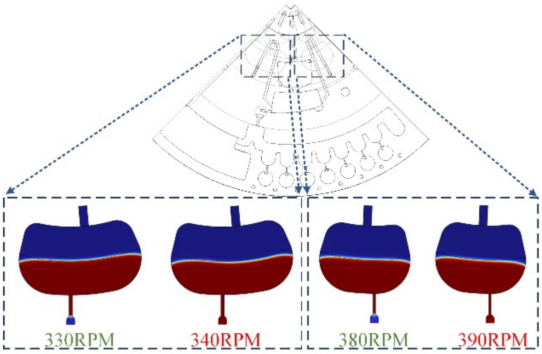

Figure S1. Differential bursting speeds of hydrophobic valves used for wash buffer and elution buffer release critical from simulation results.

The simulation results show that the trap has a burst speed of 340RPM and 390RPM, which is in line with our expectations. Since the actual hydrophobic valve is three-dimensional, we verified the feasibility of the theoretical formula for designing the trap based on simulation verification. Therefore, we followed the dimensions guided by the theoretical formulas to machine the hydrophobic valve for the centrifugal microfluidic chip.

TABLE S1. Reagent composition of 147uL LAMP cocktail

Reagent	Volume
DNTP（Solarbio）	24.5uL
LAMP buffer（NEBiolabs）	17.5 uL
MgSO4（NEBiolabs）	10.5 uL
EvaGreen® Dye（Biotium，USA）	8.75 uL
Bst 3.0 DNA Polymerase（NEBiolabs）	7 uL
ddH_2O	78.75 uL

TABLE S2. Primer sequence information for the LAMP reaction.

Primer	Sequence (5′ to 3′)
FIP	GATTGGGGCCAACTCCTACCGCCAGTCGAGCATCTCTTCAG
BIP	GTCCATTAATGCGTGGTCGTGCGAAGATGCGGACTTGCGT
F3	TTTACTGGCTTTGGTCGTCA
B3	TTTACTGGCTTTGGTCGTCA

TABLE S3. Reagent composition of 20 uL qPCR system

Reagent	Volume
RNase-Free ddH_2O	10 uL
Talent qPCR PreMix (SYBR GREEN)	7.8 uL
Forward primer	0.6 uL
Reverse primer	0.6 uL
Target	1 uL

TABLE S4. Primer sequence information for the qPCR reaction.

Primer	Sequence (5′ to 3′)
Forward primer	AGAACGGTTTGTGGTTTATCAGGA
Reverse primer	CGTCACAGCCAAAAGCCAG

TABLE S1. Reagent composition of 25 uL LAMP reaction

Reagent	Volume
DNTP（Solarbio）	3.5 uL
LAMP buffer（NEBiolabs）	2.5 uL
MgSO4（NEBiolabs）	1.5 uL
EvaGreen® Dye（Biotium，USA）	1.25 uL
Bst 3.0 DNA Polymerase（NEBiolabs）	1 uL
FIP primer	1 uL
BIP primer	1 uL
F3 primer	1 uL
B3 primer	1 uL
Target DNA	11.25 uL
